# 硼替佐米、泊马度胺、地塞米松方案治疗复发/难治性多发性骨髓瘤的疗效及安全性

**DOI:** 10.3760/cma.j.issn.0253-2727.2023.07.016

**Published:** 2023-07

**Authors:** 俊 王, 宏 许, 晓霞 初, 伟 王

**Affiliations:** 1 青岛大学附属医院血液科，青岛 266000 Hematology Department, The Affiliated Hospital of Qingdao University, Qingdao 266000, China; 2 烟台毓璜顶医院血液科，烟台 264000 Hematology Department, Yantai Yuhuangding Hospital, Yantai 264000, China

多发性骨髓瘤（MM）是一种浆细胞恶性肿瘤，尽管目前存在多种治疗方式，MM仍是一种不能被治愈的疾病，几乎所有患者均会经历复发难治阶段，新药如免疫调节剂（IMiD）、蛋白酶体抑制剂、单克隆抗体等的应用显得尤为重要[1]。泊马度胺作为第3代IMiD，尽管其化学结构与其他IMiD（如来那度胺）类似，但具有独特的抗癌、抗血管形成和免疫调节特性[2]。2013年美国食品药品管理局（FDA）批准泊马度胺用于治疗复发/难治性MM（RRMM）。泊马度胺可与多药联合发挥抗肿瘤作用，而目前国内尚无仅针对VPd方案（硼替佐米+泊马度胺+地塞米松）的大样本临床报道，本研究回顾性纳入58例应用VPd方案诱导治疗的RRMM患者，对其疗效及药物的安全性进行分析并报道如下。

## 病例与方法

1. 病例：纳入2020年12月至2022年8月于青岛大学附属医院、烟台毓璜顶医院就诊并接受至少1个周期VPd方案诱导治疗的58例RRMM患者，纳入标准如下：①符合国际骨髓瘤工作组（IMWG）及美国血液协会相关共识对“RRMM”的定义[3]–[4]；②ECOG评分≤2分，预计生存期超过3个月；③无活动性感染性疾病；④主要脏器无严重器质性病变（由本病引起的肾功能不全除外）。采集的临床数据包括：首次复发年龄、性别、ECOG评分、血常规、肝肾功能、乳酸脱氢酶、血清蛋白电泳、血/尿免疫固定电泳、血游离轻链、影像学检查、骨髓穿刺及病理检查、细胞遗传学检查等。

2. 治疗方案：患者接受VPd方案治疗，具体为：泊马度胺，口服，4 mg/d，第1～21天；硼替佐米，皮下给药，1.3 mg·m^−2^·d^−1^，第1、8、15、22天；地塞米松，口服，20 mg/d，第1、2、8、9、15、16、22、23天；每28 d为1个治疗周期。三药联合方案应用8个周期后采用泊马度胺联合地塞米松两药联合维持治疗直至疾病进展。

3. 疗效评估：治疗前和每周期治疗结束后评估疗效，根据IMWG制定的疗效评估标准对患者进行疗效评估，分为严格意义的完全缓解（sCR）、完全缓解（CR）、非常好的部分缓解（VGPR）、部分缓解（PR）、微小缓解（MR）、疾病稳定（SD）和疾病进展（PD）。总有效率（ORR）定义为sCR率、CR率、VGPR率及PR率之和。

4. 安全性评估：根据CACTE 5.0版，每周期对患者用药发生的不良事件进行分级。

5. 随访：通过查阅病历或电话随访，随访日期截至2022年8月20日。总生存（OS）时间定义为自首次应用VPd方案至患者死亡或随访终止的时间。无进展生存（PFS）时间定义为自首次应用VPd方案至患者疾病进展、死亡或随访终止的时间。

6. 统计学处理：应用SPSS 26.0软件进行数据处理。计数资料以例数（百分比）表示，组间率的比较采用*χ*^2^检验或Fisher确切概率法。计量资料以中位数（范围）表示，采用非参数秩和检验进行比较。生存曲线绘制采用Kaplan-Meier法。*P*<0.05为差异有统计学意义。

## 结果

1. 临床特征：男34例（58.6％），女24例（41.4％），首次复发时中位年龄为64（45～79）岁。首次应用VPd方案前中位WBC为3.25（1.33～11.96）×10^9^/L，中位HGB为86（67～139）g/L，中位ANC为3.67（1.65～10.02）×10^9^/L，中位PLT为211（13～414）×10^9^/L。肾功能不全患者25例（43.1％）。根据修订版国际分期系统（R-ISS）标准[5]分组（初诊时），Ⅰ、Ⅱ期患者18例（31.0％），Ⅲ期患者40例（69.0％）。有细胞遗传学资料的患者45例（77.6％），根据Mayo骨髓瘤分层及风险调整治疗（mSMART）分层系统标准分组[6]，其中高危35例（77.8％），标危10例（22.2％），两个及以上基因突变8例，高危患者中，del（17p）16例，t（4;14）10例，p53突变3例，1q扩增3例，t（14;16）2例，t（14;20）1例，因样本量受限，本研究仅对del（17p）、t（4;14）组行进一步研究。全部患者既往接受过一线治疗，其中位PFS时间为5.4个月，51例（87.9％）患者接受过二线及以上治疗，其中位PFS时间为4.1个月；全部患者均应用过来那度胺治疗，其中36例（62.1％）耐药，22例（37.9％）敏感；全部患者既往均应用过硼替佐米治疗，其中15例（25.9％）耐药，43例（74.1％）敏感；30例（51.7％）患者接受过达雷妥尤单抗治疗，其中18例敏感，12例耐药；仅5例（8.6％）患者接受自体造血干细胞移植治疗，中位PFS时间为5.8个月。

2. 疗效分析：截至末次随访时间2022年8月20日，中位随访时间9.9（4.1～20.8）个月，所有患者均完成4个周期治疗，其中3例（5.2％）达CR，8例（13.8％）达VGPR，ORR为86.2％（50/58）。40例患者完成8个周期治疗，其中7例（17.5％）达CR，11例（27.5％）达VGPR，ORR为82.5％（33/40）。21例患者完成12个周期治疗，其中8例（38.1％）达CR，12例（57.1％）达VGPR，ORR为100.0％（21/21）。分析完成12个周期治疗的21例患者的疗效，VPd方案的中位起效疗程数为2个周期，而达到最佳疗效的中位治疗周期数为5个。所有患者的中位PFS时间为10.2（2.3～20.8）个月，中位OS时间为15.8（4.5～20.8）个月，1例患者因疾病进展死亡。至本研究结束时，21例（36.2％）患者仍在治疗。37例患者因疾病进展等原因终止治疗。

根据首次复发年龄（≥60岁对<60岁）、性别（男对女）、复发次数（首次对多次）、有无髓外累及（有对无）、骨髓瘤细胞数（≥40％对<40％）、肌酐清除率（CCr）（≥60 ml/min对<60 ml/min）、R-ISS分期（Ⅰ、Ⅱ期对Ⅲ期）、细胞遗传学分层（标危对高危）分组进行比较，PFS和OS的差异均无统计学意义（*P*值均≥0.05）。来那度胺耐药、敏感患者的中位PFS时间分别为12.7个月和8.1个月，1年PFS率分别为33.3％（95％*CI* 20.0％～59.9％）、54.5％（95％*CI* 35.7％～79.8％），差异有统计学意义（*P*＝0.044），两组的PFS曲线见图1。因细胞遗传学高危患者的样本量有限，本研究仅针对del（17p）和t（4;14）两种突变进行进一步研究，del（17p）、t（4;14）患者的中位PFS时间分别为11.6个月和6.8个月，1年PFS率分别为43.8％（95％*CI* 22.7％～60.8％）、30.0％（95％*CI* 19.5％～52.1％），差异有统计学意义（*P*＝0.015），两组的PFS曲线见图2。

**图1:**
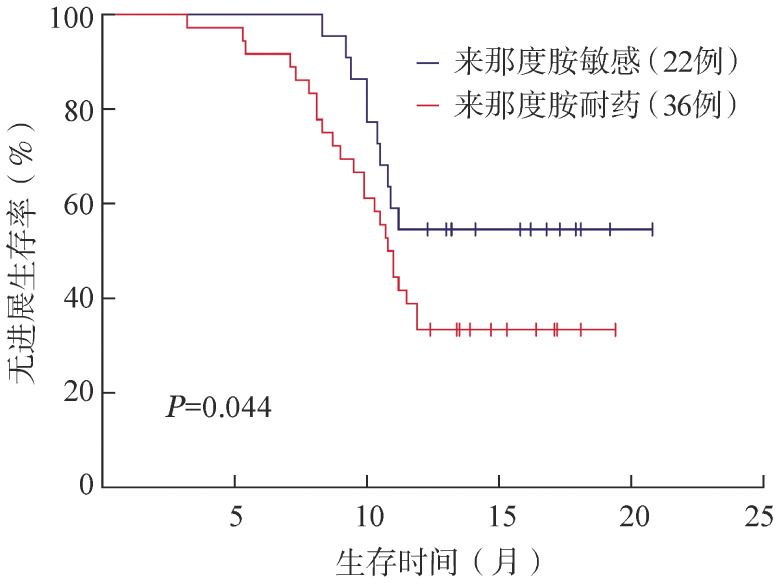
来那度胺敏感、耐药的复发/难治性多发性骨髓瘤患者的无进展生存曲线

**图2:**
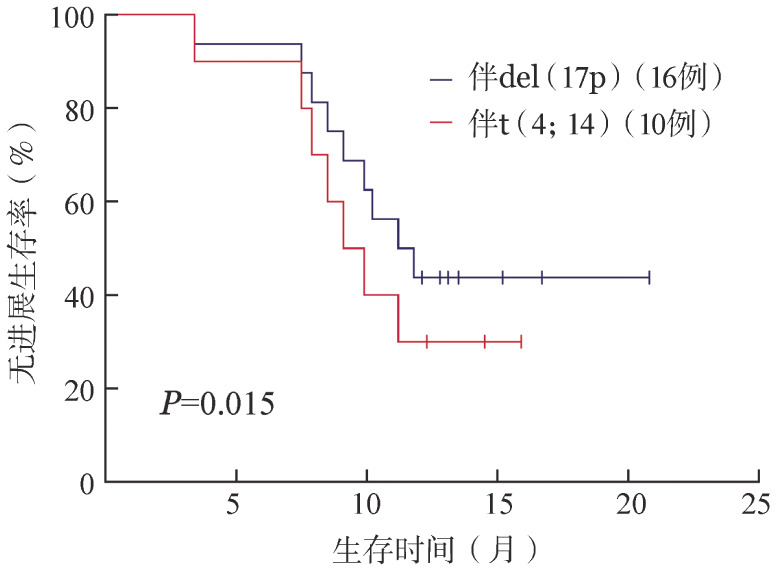
伴del（17p）、t（4;14）的复发/难治性多发性骨髓瘤患者的无进展生存曲线

对25例肾功能不全患者的肌酐（Cr）及CCr进行分析，12个疗程治疗后Cr较治疗前基线水平明显降低［76.8（41.9～176.0）µmol/L对111.5（51.7～299.1）µmol/L］，差异有统计学意义（*P*＝0.039）；CCr水平明显升高［71.4（43.9～108.5）µmol/L对65.3（33.5～93.7）µmol/L］，差异有统计学意义（*P*＝0.024）。治疗8个疗程与4个疗程相比，患者Cr水平降低［75.4（49.3～208.4）µmol/L对81.7（46.1～237.9）µmol/L］，差异有统计学意义（*P*＝0.029）；CCr水平升高［75.2（38.7～97.1）ml/min对69.9（40.0～128.6）ml/min］，差异有统计学意义（*P*＝0.042）。治疗12个疗程与4个疗程相比，Cr、CCr的差异均无统计学意义（*P*值均>0.05）。25例肾功能不全的患者中，治疗前CCr<60 ml/min的患者22例，治疗后CCr上升至≥60 ml/min且可维持1个月以上者被认为是肾功能逆转，4个周期后15例（68.2％）患者肾功能逆转。

3. 安全性分析：所有患者均接受安全性评估，发生的不良事件（AE）包括血小板减少12例（20.7％）、中性粒细胞减少10例（17.2％）、贫血5例（8.6％）、肺部感染1例（1.7％）、带状疱疹1例（1.7％）。3例患者发生3～4级AE，均为血小板减少。血液学AE经过治疗均可缓解，肺部感染、带状疱疹经抗生素、抗病毒治疗后好转，治疗过程中未出现因AE死亡或泊马度胺减量的情况。

## 讨论

RRMM的治疗选择应考虑前期治疗药物、疗效、相关不良反应并对疾病当前状态进行预后因素评估等[7]。泊马度胺通过对微环境的影响（如调节免疫反应、抑制血管生成、抑制骨吸收等）诱导骨髓瘤细胞凋亡，并产生间接抗骨髓瘤效应[2]。泊马度胺可与多药联用发挥抗骨髓瘤作用，MM-003Ⅲ期研究显示，泊马度胺联合低剂量地塞米松（P+Lo-Dex）方案的中位PFS时间为4.0个月，联合高剂量地塞米松（P+Hi-Dex）方案的中位PFS时间为1.9个月[8]。AMN001Ⅱ期研究显示，泊马度胺、环磷酰胺、地塞米松（PCd）方案的中位PFS时间为9.0个月[9]。Krishnan等[10]进行的Ⅰ～Ⅱ期研究中，伊沙佐米、泊马度胺、地塞米松（Ixa-Pd）方案的中位PFS时间为8.6个月。Bringhen等[11]进行的Ⅰ～Ⅱ期研究中，卡非佐米、泊马度胺、地塞米松（KPd）方案的中位PFS时间为10.3个月。APOLLO Ⅲ期研究中，达雷妥尤单抗、泊马度胺、地塞米松（Dara-Pd）方案的中位PFS时间为12.4个月[12]。ICARIA-MM Ⅲ期研究显示，伊沙妥昔单抗、泊马度胺、地塞米松（Isa-Pd）方案的中位PFS时间为11.5个月[13]–[14]。OPTIMISMM Ⅲ期临床研究显示，VPd方案的中位PFS时间为11.2个月。可见在泊马度胺联合其他药物治疗RRMM的不同方案中，VPd方案有较好的疗效，但针对此方案的国内大样本临床研究仍缺乏，故本研究分析了VPd方案对RRMM患者的疗效，并在此基础上进行分组，进一步研究各因素对疗效的影响[15]–[16]。

本研究中所有患者的ORR为86.2％，中位PFS时间为10.2个月，反应率与OPTIMISMM Ⅲ期临床研究相似[15]–[16]。本研究纳入来那度胺耐药患者，耐药组及敏感组的ORR分别为83.3％、90.9％，1年PFS率分别为33.3％、54.5％，且单因素分析结果显示差异有统计学意义，显示既往应用来那度胺诱导治疗且耐药的RRMM患者应用VPd方案治疗的疗效较来那度胺敏感者差，与OPTIMISMM Ⅲ期临床研究[15]–[16]的结果相近，但本研究及既往临床研究中两组ORR均较高，因此，VPd方案仍可作为该部分患者复发后的治疗选择。

RRMM患者通常伴高危细胞遗传学特征，随着新药的研发及广泛运用，MM患者的预后得到显著改善，但高危细胞遗传学患者的预后仍然不佳[17]。有研究发现，Pd方案可部分克服高危细胞遗传学的不良预后，特别是对于伴del（17p）、t（4;14）的患者[18]。本研究中，高危组患者各疗程的ORR与标危组相当，两组PFS率的差异无统计学意义，受样本量影响，本研究仅比较了del（17p）和t（4;14）患者的疗效，两组ORR分别为87.5％、80.0％，1年PFS率分别为43.8％、30.0％，单因素分析结果显示差异有统计学意义。可见VPd方案对于伴del（17p）的RRMM患者显示良好的疗效，对于伴t（4;14）的患者疗效稍差，但ORR水平尚可，患者仍可获益，本研究中两组患者样本量较少，该结论有待扩大样本量后进一步研究。

肾功能不全是MM常见的并发症，伴肾功能损害的MM患者病死率高、生存期短[19]–[20]。本研究中，CCr<60 ml/min患者的ORR与CCr≥60 ml/min患者相当，两组1年PFS率的差异无统计学意义，此结果可能受到应用硼替佐米及入组患者肾功能损伤程度较轻等因素的影响。而在25例肾功能不全的患者中，治疗前CCr<60 ml/min的患者22例，VPd方案治疗后肾功能逆转率为81.8％，与治疗前CCr相比，治疗后患者的CCr升高，差异有统计学意义，表明VPd方案可能克服肾功能不全对RRMM患者预后的影响。

多中心Ⅱ期临床试验显示，泊马度胺相关方案AE发生率较高，但3～4级AE发生率低[21]，最常见的AE包括白细胞减少、中性粒细胞减少、血小板减少、贫血、感染等。本研究中血液学AE主要为血小板减少、中性粒细胞减少，非血液学AE主要为感染，3例患者发生3～4级AE，均为血小板减少。发生AE的患者经对症治疗后均可缓解，治疗过程中未出现因AE药物减量、停药或死亡的患者。与多中心临床试验相比，本研究中AE发生率较低，原因可能是入组患者均为RRMM患者，入组时已存在上述不良反应，在应用VPd方案的同时已予对症处理，可能在一定程度上减轻药物的不良反应，由此可说明VPd方案治疗相关不良反应大多可耐受、逆转，总体安全性良好。

综上，本研究结果提示VPd方案治疗RRMM患者具有良好的疗效及安全性，并且可在一定程度上改善肾功能。对于伴del（17p）的患者，VPd方案显示出了良好的疗效；对于来那度胺耐药的患者，VPd方案也可获得一定的疗效，仍可作为治疗选择。但本研究样本量较少，部分患者随访时间较短，未达到中位OS时间，尚待进一步扩大样本量及延长随访时间加以验证。
